# “All I want is to be involved in my treatment”: Experiences of HIV treatment and viral load monitoring among adolescents, young adults and healthcare providers in Tanzania

**DOI:** 10.1371/journal.pone.0320272

**Published:** 2025-06-25

**Authors:** Joan Rugemalila, Anna Minja, Magreat Somba, Adellah Sariah, Hellen Siril, Christopher Sudfeld, Tumaini Nagu, Bruno Sunguya, Said Aboud

**Affiliations:** 1 Department of Microbiology and Immunology, Muhimbili University of Health and Allied Sciences, Dar es Salaam, Tanzania; 2 Department of Internal Medicine, Muhimbili National Hospital, Dar es Salaam, Tanzania; 3 Muhimbili University of Health and Allied Sciences, Dar es Salaam, Tanzania; 4 Kairuki University, Department of Mental Health and Psychiatric Nursing, Dar es Salaam, Tanzania; 5 Management and Development for Health, Dar es Salaam Tanzania; 6 Harvard T. Chan School of Public Health, Massachusetts, United States of America; 7 Department of Internal Medicine, Muhimbili University of Health and Allied Sciences, Dar es Salaam, Tanzania; 8 Department of Community Health, Muhimbili University of Health and Allied Sciences, Dar es Salaam, Tanzania; South African Medical Research Council, SOUTH AFRICA

## Abstract

**Background:**

Virologic response is the earliest indicator of HIV treatment, making it the gold standard in monitoring treatment response. This study explored experiences and challenges of HIV treatment and viral load (HVL) monitoring among **adolescents and young adults (AYA), parents or guardians of young adolescents** and healthcare providers (HCPs).

**Methods:**

This was a qualitative study conducted between March and May 2022 in five health facilities in an urban setting of Tanzania. We performed 20 in-depth interviews (IDIs) with AYA who had full HIV-positive status disclosure, 8 IDIs with parents or guardians of young HIV-infected adolescents, and five focus group discussions (FGDs) with 30 HCPs. The IDIs and FGD transcripts were translated from Swahili into English, transcribed, coded, and performed thematic analysis using NVivo software.

**Results:**

AYA demonstrated an understanding of HIV treatment as lifelong and, most knew the benefits of HIV viral load (HVL) monitoring. It was apparent that 60% of older adolescents (15–19 years), parents or guardians, and young adults (20–24 years) discussed HVL results with HCPs and, the majority desire to be involved in their HIV treatment. HCPs reported that missing clinic appointments among AYA attending boarding schools or college contribute to delays in HVL testing and initiation of **enhanced adherence counselling** (EAC). Notably, HCP faced challenges recalling AYA who received three to six multi-month **anti-retroviral drug (ARV)** refills when their HVL results were high (VL ≥ 1000 copies/ml).

**Conclusions:**

More than half of AYA expressed ownership in their HIV treatment; they discussed the meaning of their HVL results with HCPs. Additionally, AYA reported non-frequent challenges in HVL testing however, HCPs described missing appointments for AYA on three to six multi-months dispensing of ARVs as major challenges for implementing HVL testing. Importantly multi-months ARV dispensing in AYA needs program evaluation to inform the best implementation modality to support viral suppression.

**Key findings**

- Most adolescents and young adults living with HIV are aware that antiretroviral therapy (ART) is a lifelong treatment, and some desire to be fully engaged in their HIV treatment.
- Generally, adolescents, young adults and their parents or guardians understand that viral load testing is essential for monitoring the effectiveness of HIV treatment.
- A low viral load provides encouragement and motivation to adolescents and young adults , a sign that their treatment is effective. Conversely, a high viral load can lead to feelings of disappointment and even despair.
- Healthcare providers follow national guidelines for HIV viral load testing, but most adolescents miss scheduled viral load testing, especially with 3–6-month ARV refills.

## Introduction

Globally the number of adolescents living with HIV aged 10–19 years is estimated at about 1.8 million, out of these more than 80% are found in sub-Saharan Africa (SSA) [1]. **A significant burden in HIV epidemic in Tanzania is among young people particularly girls and young women [2]. Despite increasing access to antiretroviral therapy (ART) among HIV-infected adolescents in SSA, the desired target of low levels of viral suppression and a decline in HIV-related deaths have not been achieved particularly in Eastern and Southern Africa** [3,4]. Adolescents receiving ART in sub-Saharan Africa (SSA) continue to experience poor virologic outcomes posing a challenge to controlling the HIV epidemic and threatens the achievement of the third UNAIDS 95 target which focuses on viral suppression among 95% of PLHIV receiving ART [5,6].

HIV viral load (HVL) monitoring refers to the regular **measurement of the amount of the HIV virus** in blood. **This recommended approach enables early detection of ART response and is the most reliable indication of treatment failure for the timely switch to effective ART** [6]. In resource-limited settings, there are reports of delays in switching ART regimens due to inadequate viral load monitoring [7,8]. **Furthermore, in low- and middle-income countries, despite the scale-up of centralized HIV viral load (HVL) testing to improve access, challenges in sample transportation systems, shortage of human resources, and financial limitations have undermine the full implementation of HVL testing** [9,10]. In addition, individual factors affect routine HVL monitoring uptake, including but not limited to the age and duration of ART [10,11].

In 2016, Tanzania adopted the world health organization (WHO) guidelines recommending that all PLHIV must undergo HIV viral load testing 6 and 12 months after ART initiation. Thereafter, at 12 months, PLHIV who have achieved viral suppression will receive an annual HIV viral load test. Viral suppression is defined as HIV viral load <1000 copies/ml after six months of ART [12,13]. PLHIV with unsuppressed viral load (VL ≥ 1000 copies/ml) are provided with **enhanced adherence counselling (EAC),** and after 3 months a repeat viral load test is done to exclude or confirm virologic failure. Importantly, optimal HVL monitoring will enable PLHIV to stay longer on a first-line ART regimen, maximize the benefit of treatment as prevention of HIV transmission and prevent the development of acquired drug resistance.

Strengthening HIV treatment monitoring (particularly HVL monitoring) to improve VS in AYA is important because HVL monitoring provide treatment guidance, and monitor patient response to ART. Thus, it is essential to understand the implementation status from both recipients of care (AYA) and the providers (HCPs) of HVL monitoring to better understand the reported lower HVL monitoring uptake. **In the context of test-and-treat strategy and the scale-up of HIV viral load monitoring in Tanzania, this study aimed to explore experiences with HIV treatment and HVL monitoring among AYA who are aware of their HIV status, healthcare providers and parents or guardians of young HIV-infected adolescents aged 10–14 years.** The findings will inform what needs to be done for AYA during the cascade of care and, for HCPs as they utilize HIV viral load monitoring guidelines to maximize uptake and improve ART outcomes in AYA. Importantly, our study findings will highlight gaps or successes in the national ART program focusing on adolescent ART services.

## Materials and methods

### Study design

We conducted a phenomenological study using IDIs and FGDs in five care and treatment (CTC) facilities in Dar es Salaam, Tanzania, between March 2022 and May 2022. IDIs were conducted amongst AYA with full HIV-positive status disclosure and parents or guardians accompanying young adolescents (10–14 years) to care. Importantly, IDIs are the appropriate method in the setting that aims to explore information from young people who may face the challenges of stigma. Additionally, a one-to-one IDI provides privacy after building rapport between the investigator and interviewee; this makes an individual comfortable discussing HIV- related issues with an investigator rather than in a group [14]. Finally, we chose FGDs because it is an approach that promotes interaction among participants allowing social interaction of the group, maintaining group norms providing in-depth information to understand groups’ perspectives in terms of collective conclusions [15,16].

### Study settings and population

**Our study was conducted in Dar es Salaam region which in 2022 had a regional HIV prevalence of 4.2% and, the overall national prevalence was 4.5% among PLHIV aged 15 years and above** [17] **with the highest number of PLHIV enrolled in HIV care** [18]. Dar es Salaam has a total number of 228 facilities offering ART services (159 dispensaries, 36 health centers, and 33 hospitals); out of these 49% are public and 28.5% are private, 18.4% faith-based organization and 6% military facilities. All health facilities (public and private) are categorized as dispensaries, health centers, district hospitals, regional referral hospital hospitals at zonal level and tertiary hospitals such as national hospitals. **Both private and public health facilities follow the standard HVL monitoring outlined in the Tanzania national guidelines for the management of HIV and AIDS of 2019** [19]. Notably, AYA living with HIV are estimated to be 13,776 in Dar es Salaam reported in the District Health Information System (DHIS-2). In 2017, the Tanzania Health Impact Survey (THIS) **reported** an overall VS estimated at 29.8% among adolescents aged 15–19 years, and 71.9% of those aged 20–24 years reported having tested and received their results [17]. However, Dar es Salaam region VS was not reported due to minimal number (less than 25) of unweighted cases had been suppressed and therefore, VS rate for AYA has not been documented for this age group. **The absence of data from the Dar es Salaam region creates a significant gap in understanding how adolescents and young adults (AYA) in this area are responding to HIV care and treatment interventions.**

Dar es Salaam has two centralized HVL testing laboratories, Temeke specialized laboratory supported by Management and Development for Health (MDH) a non-governmental organization, and Muhimbili National Hospital. These serve to perform HVL testing for samples from within the Dar es Salaam region as a hub and also for outside Dar es Salaam to send HVL samples for testing.

## Ethical considerations

This study received approval from National Research Ethics Committee of the National Institute for Medical Research in Tanzania (NIMR/HQ/R.8a/Vol.IX/4418 and Muhimbili University of Health and Allied Sciences Institutional Review Boards (MUHAS-REC-01-2022-94).

Before participating in IDIs or FGDs, all our study participants were informed of the study procedure and thereafter those who agreed were requested to provide a signed written informed consent. Notably, we obtained written informed consent from participants aged 18 and above years, assent from 15–17 years together with a parent or legal guardian informed consent and, for minors aged 10–14 years a written informed consent from a parent and/or legal guardian. No identifiable information of the study participants has been included during data collection, analysis and presentation of findings. All the methods included in this study are in accordance with the declaration of Helsinki.

### Recruitment and data collection

The study team piloted the data collection tools for IDIs and FGD guides. The interview guides were piloted by the PI and one investigator (AM) and at one purposefully selected CTC facility in Ilala district, Dar es Salaam. This facility was not part of the study sites and involved 8 participants with our study eligibility criteria; 4 adolescents aged between 15 and 19 years and 4 HCPs. This pilot aimed to observe the understanding of the interview questions regarding clarity, language, and the average time it took for each participant to complete the interview. The study team used the pilot findings only to make necessary changes to improve the interview guides for IDI and FGD. Two social scientists (AM and MS) used the revised guides to train other three investigators before beginning data collection. After that, ART nurse or care and treatment nurse in charge contacted potential participants using mobile phone calls to remind AYA of their usual clinic schedule and request participation in the study. Thereafter, two study investigators obtained informed written assent and/or consent from AYA after finishing their clinic visit. We obtained written informed consent from participants aged 18–24 years and assent from 15–17 years with parent or guardian consent. Recruitment involved a purposeful selection of participants who have been on ART for > 6 months or more and have had full HIV status disclosure. The IDIs were done during the adolescent and youth clinic days after going through health education sessions and clinician consultation.

We conducted interviews in a selected room at the study site which favored confidentiality and privacy. The main author in collaboration with three social scientists experienced with qualitative HIV-related research in people living with HIV conducted the interviews in Swahili a national language using a semi-structured interview guide in both the FGDs and IDIs. The scientists were oriented on the study and the interview guide in a 1-day session which included role-play interviews to ensure they built an adequate understanding of the study questions before the actual data collection began. The investigators alternatively interviewed AYA, parents, or guardians of young adolescents or took interview notes, and observed non-verbal communication during IDIs and FGDs. Importantly, IDI and FGDs were conducted after the participants received their routine services. Study participants were informed of the tape recording during the interview and focus group discussion. Two to three IDIs were done at one study site per day. Potential FGD participants agreed to be available on a Saturday clinic which did not have a heavy workload. Each FGD consisted of 6–8 participants depending on the available number of staffs at each site. All interviews lasted between 45 and 60 minutes and were tape-recorded. All participants received a monetary incentive equivalent to Tsh 15,000/= to compensate for their time and transport.

The themes discussed in IDIs included ART adherence and lifelong treatment, experience with HIV disclosure and ART adherence, awareness of HIV viral load monitoring, and barriers and challenges to viral load testing. Importantly, the main focus was on experiences with the implementation of viral load monitoring among AYA in this setting.

### Interview guides

The investigators developed an interview guide for IDI and FGDs focusing on the experiences of HIV treatment monitoring among AYAs after ART initiation. We used a socioecological framework that encompasses the interactive effects of intrapersonal, interpersonal, environmental, and policy factors and how these affect the well-being of a population [20]. We developed interview questions and discussed them with all study team members to make sure all interviewers have the same understanding and interpretation. The interview questions were open-ended exploring experiences of HIV viral load testing implementation on AYA receiving ART services.

In-depth interviews and focus group discussions were collectively conducted by our research team (JR, AM, MS, and AS) fluent in Swahili and English, with experience in qualitative interviews, and HIV care, the same team-led data collection and analysis. Specifically, two investigators AM and MS are trained social scientists with over 10 years of experience collecting qualitative data led the IDIs and the FGDs to minimize leading participants to answers that the researcher wants. We performed 18 IDIs with AYA receiving ART services who have full HIV status disclosure because partial or non-disclosed AYA would not be suitable to answer the study questions to explore their overall understanding of ART as a lifetime treatment and HIV viral load monitoring. In addition, 10 IDIs were also conducted among parents or guardians of 10–14 years adolescents. Notably, IDIs were performed among participants who could provide assent (15–17 years), and parent or guardian permission, and consent from AYA aged 18–24 years. Our IDI study sample included AYA with different characteristics (suppressed and unsuppressed, peer educators). The study investigators recognized the same pattern of responses and data saturation was considered after interviewing 16 AYA and eight parents or guardians and confirmed after adding four more AYA. Due to the limited availability, we could not add more parents or guardians. Importantly, study investigators agreed that thorough information was obtained which was enough to fully understand and explain the phenomenon of interest. All interviews were conducted smoothly between 50 minutes to 60 minutes for each participant after receiving their services for that day.

### Focus group discussions

We conducted FGDs among healthcare providers after they finished their work for that particular day and each discussion took an average of 60 minutes. The HCP were particularly asked questions regarding the challenges and barriers during the implementation process of HIV viral load monitoring in AYA. The focus group guide questions were based on the Social Cognitive Theory (SCT) to elicit responses concerning the implementation of HIV treatment monitoring in AYA [21]. The SCT provides a mechanism of understanding how individuals learn cognitively and influencing their behavior from observing the behavior of others and the consequences of those actions. Therefore, by applying SCT it is possible to understand the experiences of HCPs in implementation of HVL monitoring. Importantly, the FGD questions had the following SCT constructs: (1) knowledge and capability of HCP to perform HIV treatment monitoring as per national guidelines (2) HCP observing consequences of AYA’s behavior on expected health outcomes such as viral suppression, improved survival and reduced opportunistic infections and (3) experiences in performing of viral load testing when barriers or challenges are present.

Importantly, each FGD transcript was sent to the investigators who read and provided feedback to the social scientists (AM and MS) to ensure they understood the questions and allowed open discussion and expression of participants’ experiences with adequate follow-up questions and probing. Data saturation was reached after reviewing responses from 28 respondents from five FGDs and confirmed that no new information arose from participants from all study sites as they shared similar experiences thus providing similar answers. Using verbatim, the study investigators (JR, AM and MS) transcribed the tape-recorded interviews to obtain Swahili transcripts and later translated them into English. JR, AM and MS are conversant with Swahili and English languages.

### Data analysis

Data analysis began during data collection and continued throughout the process of interviews [22]. All the tape-recorded interviews were transcribed and obtained Swahili transcripts that were later translated into English by MS and JR who are all conversant with Swahili and English languages. After that, JR, AM and MS reviewed the transcripts compared to the tape records ensuring that no information is missed first in IDIs and later FDGs. We adopted thematic analysis because first, the investigators wanted to read through collected data and searched for collective understanding of what is HVL monitoring, its importance and interpretation in lifelong HIV treatment within AYA and, secondly in HCPs by examining who says what about her/his experiences and made inferences about the messages. Overall, our main goals in this analysis was to identify and understand themes, explore how the qualitative data can inform theoretical claims made in research studies such as reports of a lower uptake below 50% of HVL testing and non-uptake of HVL testing in younger age [23–25]. Using three phases of content analysis processes: preparation, organization, and reporting of results, the authors analyzed and quantified the data [22]. The main author read through the data and identified the coding units and apply the coding units in sorting the data into categories by grouping codes related to each other. We used NVivo software during the inductive approach in the organization phase performing open coding, later creating categories, and emerging main themes. All investigators discussed the study findings to ensure quality and valid coding and analysis.

### Trustworthiness of the study

We declare that our study findings are trustworthy because we applied the constructs of trustworthiness [26]. We used the triangulation method for credibility using two sources of information; interviewing participants and observation during data collection until the investigators reached saturation (a repeated pattern of information). In addition, investigators performed data triangulation during IDIs and FDGs, which focused on implementing HIV treatment monitoring. Also, investigators checked participants’ responses during face-to-face interviews to ensure adequately captured responses to critical questions were in the audio recording and documentation of field notes. Notably, the principal investigator is an experienced Infectious disease physician working with transitioning adolescent HIV care to adult care with HIV treatment monitoring challenges in the Tanzanian setting. Additionally, the co-investigators have vast experience conducting IDIs and FGDs in HIV care settings among PLHIV in Tanzania. Our study investigators JR, AM, MS and AS performed debriefing of data collected, performed transcription and joint analysis to ensure. Another researcher (HS) experienced with qualitative research in HIV populations was requested to read and react to the study findings and interpretation analysis before publishing to fulfil dependability as part of trustworthiness.

## Results

### Participants characteristics

**Sixty-four percent of in-depth interviewees were female adolescents aged 10–19 years, while the remaining participants were all female young adults aged 20–24 years.**

Overall, 39.3% of IDI participants were attending school or college, 28.3% were married and 75% were living with a family member who is living with HIV. Study participants aged 15 years and above had disclosed their status to their sexual partners or family members. The characteristics of IDI participants are presented in Table 1. On the other hand, the age of HCPs ranged from 26–58 years, about 26.6% and 23.3% had diploma in nursing and clinical medicine respectively. Forty percent of HCPs had experience in providing HIV care services for more than 10 years and, 36.6% had working experience with adolescents of more than 10 years as shown in Table 2.

**Table 1:** Demographic Characteristics of Adolescents and Young Adults (AYA) and Parents/Guardians of Adolescents Aged 10-14 Years Enrolled in In-Depth Interviews (n = 28).

Variable	Frequency (N)	Percentage (%)
**Age of Participants**		
parents or guardians of 10–14 years adolescents	4	14.3%
15-19 years	9	32.1%
20-24 years	6	21.4%
≥25 years	9	32.1%
**Sex of Participants**		
Male	10	35.7%
Female	18	64.2%
**Marital Status**		
Single	19	67.9%
Married	8	28.3%
Widowed	1	3.6%
**Level of Education**		
No formal education	4	14.3%
Primary education	9	32.1%
Secondary education	13	46.4%
College education	2	7.1%
**Occupation**		
None	9	32.1%
Attending school/college	11	39.3%
Business	6	21.4%
Mechanic	1	3.6%
Peer educator	1	3.6%
**HIV Status Disclosure to Family Members**		
No	3	10.7%
Yes	25	89.3%
**Living with a Family Member with HIV**		
No	7	25.0%
Yes	21	75.0%

**Table 2:** Demographic Characteristics of Healthcare Provider Participants in Focus Group Discussions (N = 30).

Variable	Frequency (N)	Percentage (%)
**Age of Participants (years)**		
≤25 years	2	6.6%
26-45 years	18	60.0%
>45 years	10	33.4%
**Gender**		
Male	9	30.0%
Female	21	70.0%
**Level of Education**		
Certificate in Social Work	2	6.6%
Certificate in Nursing	4	13.3%
Diploma in Nursing	8	26.6%
Diploma in Clinical Medicine	7	23.3%
Diploma in Pharmacy	2	6.6%
Advanced Diploma in Clinical Medicine	5	16.6%
Degree in Social Work	4	13.3%
**Experience in Providing HIV Care Services (years)**		
<2 years	3	10.0%
2-5 years	7	23.3%
6-10 years	9	30.0%
>10 years	12	40.0%
**Duration of Providing Care to AYA (years)**		
<2 years	2	6.6%
2-5 years	11	36.6%
6-10 years	7	23.3%
>10 years	11	36.6%

We present our focus group discussion findings into three main themes and seven categories as shown in Table 3.

**Table 3:** Categories and Themes Identified during Focus Group Discussions.

SN	Themes	Categories
**I.**	ART Adherence in HIV Lifelong Treatment	• Opinions on ART regimen or type
		• Treatment fatigue
		• HIV status disclosure
**II.**	Understanding HIV Treatment Monitoring	• Information on HIV viral load testing
		• Interpretation of viral load results
**III.**	Barriers or Challenges to Viral Load Testing	• Individual involvement in testing
		• Challenges with centralized viral load testing

### Theme 1: ART adherence in HIV lifelong treatment

Most IDI participants were aware that ART is a lifelong treatment. One adolescent who was a single mother stated that she is aware of the necessity for lifelong treatment and that she must continue taking ARVs to maintain her health so she can care for her only child. On the other side, few participants claimed that if they keep taking their prescribed medications, the virus will eventually be eradicated.

***“*** *I understand that we are using medications not for cure. I did not know at the beginning about viruses but later I discovered that the viruses sleep and they do not die. So, they told me they sleep but if you don’t take medications they wake up. (IDI respondent no 4; 17-years female)*

One youth reported that ART is good to maintain her health since she was a child until now feeling healthy.

*“My perception is that ARVs are good and helpful since I was a child until today, I am good and nobody knows anything because I look okay and going to school. Therefore, ARVs are good and very helpful. (IDI respondent no 19; 17-years female)*

Overall, AYA were aware that the test results to monitor HIV treatment will not be good as a result of poor adherence. Sixty-seven percent of IDI respondents stated that when a person takes medication correctly, the viral load will be low, whereas non-adherence causes the virus to multiply and increase, allowing for opportunistic infections and poor health.

Two of the participants in our study who work with peers as peer educators said that when they don’t receive support and encouragement from their families, school teachers, or some religious teachers, most adolescents and youth struggle with adhering to their medications.

*“There was religious teacher who changed the time of our classes, instead of leaving at six pm, he released us at seven pm. So, I delayed to arrive home and therefore, the time for medicines passed, I didn’t take. This new teacher is very harsh, he is not listening, and if you tell him something he beats you instead of listening” (IDI respondent no 8; 12-years male)*

Parents and guardians reported the need to supervise young adolescents (less than 15 years) because many of them do not like medicines. In addition, parents have witnessed health problems in children with non-adherence therefore, it is mandatory to ensure their child is taking ARVs on time.

*“Aa, at work is very tight and most days I go back home late night and the time for my child to take ARVs is at 6pm. So, after his blood test results I was told by her aunt that the child was not taking medications, he used to throw the pills outside” (IDI respondent no 22; 47-years male)*

Similarly, HCPs described that they experience some children and adolescents become non-adherent to ARVs due to unfriendly family environment. In addition, HCPs reported that, about a quarter adolescent despite full disclosure, they have not accepted their HIV positive status, questioning; why their peers don’t have the disease, how they got the virus or blaming their parents and become non-adherent.

“*Sometimes parents or guardian do not make close supervision of the adolescent and they usually become non-adherent to medications, so we have to call a parent or guardian and talk to him/her about supporting the child or adolescent to take their ARVs correctly. (FGD* *respondent no 18; 47-years female, nurse counsellor)”*

*“Some adolescents do not accept the HIV condition they ask how they got it or why the mother is not taking the medicine. For adolescents taking medicines is something awful, they don’t like it. (FGD respondent no 3; 56-years female, nurse counsellor)*

### Category I: Opinion on ART regimen or type

AYA and parents or guardians have accepted taking lifetime ARVs however, three adolescents proposed getting injections rather than daily pills because they believed injections would be easier to adhere to their regimen.

An older adolescent explained how she wishes to get injectable ARVs:

*“The medicine is good but the government should help us one thing for now if possible they should help us with injection*. *If you get injection, you stay for six or three months and when you visit again clinic you test to check the progress. So, we would like the government to change and give us injections instead of pills as we heard there are injections discovered” (IDI respondent no 6; 19-years female)*

Notably, ART adherence challenges were reported in young adolescents who are prescribed twice-daily medications, but a once-daily dose improves adherence.

One adolescent and one youth expressed that:

*“It is better one pill which decreases the load of having many pills and since you take it once it is better because you can plan well your time for medicines than taking twice, these are not friendly to be taken on time. (IDI respondent no 15; 16-years female)*

*I may complete a day without taking medicines because of work. So, I miss the morning dose or sometimes the evening dose if finish work late and when I come for follow up, I get poor results. So, not only the doctor but also all of the nurses become disappointed when they hear about my poor results. (IDI respondent no 10; 24-years female)*

### Category II: Treatment fatigue

Some of adolescents have agreed to the HIV treatment, but they are tired and want the illness to end. One participant expressed a concern regarding lifelong medications may cause some body side effects in the future. Similarly, during FGDs, HCPs described that most of the AYA are tired of taking daily medications, some accept the situation but many expresses challenges taking ARVs every day.

Another adolescent expressed fatigue in taking ARVs:

*I don’t like it [ARVs] very much; I wish for it to be over even today or tomorrow. I hate it, I hate it.* *I am already tired, I have been taking drugs since standard three (8 years), every day’s drugs, drugs and drugs and now I’m tired of these drugs. (IDI respondent no 4; 15-years female)*

*HCP explained the following:*

*We may get a child of 10 or 12 years who comes alone and says they are tired of taking medicines every day. You will talk to him/her to help them accept the situation and assess for adherence for you to see his/her medications, you see that the medicines do not match, meaning they skip taking them. FGD respondent no 4; 58-years female nursing assistant)*

### Category III: HIV status disclosure

Most of AYA and parents or guardians living with HIV have disclosed their HIV status to other family members, including their parents, siblings, grandparents, aunts, and uncles. Only two youth had not disclosed their HIV status to anyone. Importantly, the experience of non-disclosure to family members with whom the client lives with was found to affect ART adherence. One adolescent reported that currently his stepfather does not know his status; in that case, he is not free to take ARVs, and another adolescent stopped taking ARVs when her family received relatives to stay with them. Another adolescent also reported that they could not take their pills when their college fellows were present.

An older adolescent expressed:

*“I stopped medication because there were visitors at home place who came to stay with us for some time and we were sharing a bedroom. (IDI respondent no 1; 16-years female)*

Similarly, one youth said;

*“I cannot take the medications inside a shared bedroom, my siblings from a step father will see me” You can take paracetamol in front of a person but you cannot take these medications in front of people. So, you either wait for them to leave or if they haven’t left, then that day is going to pass” (IDI respondent no 9; 17-years female)*

### Theme 2: Understanding of HIV treatment monitoring

**AYA and parents or guardians shared a common understanding of HVL monitoring. They acknowledged its role in evaluating the effectiveness of HIV treatment and reflecting adherence to ART. AYA, in particular, pointed out that viral load testing provides a clear view of their HIV treatment progress and guides clinical decision-making.**

**Parents or guardians also reported that they usually follow up by asking HCPs about the details of the HVL results of their children or young adolescents. The young adults knew that if the viral load results showed the virus had decreased, it would enable them to receive a six-month ARV dose. Therefore, when the results are good, they feel inspired, knowing the virus is controlled, and are motivated to continue with medications correctly. This inspiration and motivation are key to maintaining a positive outlook on HIV treatment.**

However, few young adult participants with poor HVL results lost hope because they believed they were properly taking their medicine.

One adolescent explained that:

*“They monitor to check how you are taking your medicine because, if you have good adherence to medication you will control the virus they will not increase, they become low and if a person has poor adherence the test results will show a high viral load” (IDI respondent no 3; 16-years male)*

Another two adolescents expressed the following:

*“You know when you are told that your progress is good, it gives you peace of mind, work on your dreams and it means the medicine you take are working” (IDI respondent no 8; 17-years female)*

*“When I visit here after every six months and take blood for check-ups. I will know if the medicines I am using are working in my body or not working” (IDI respondent no 20; 12-years male)*

One youth reported disappointment but had self-encouragement:

***“*** *When the results are bad, they hurt you because you are taking the medications and they are not working. You will think, if there is somewhere somehow you went wrong you take the chance to improve yourself”. (IDI respondent no 15; 21-years male)*

One parent expressed eagerness to know treatment progress of her child:

*“It is good to know treatment progress, if they check it today when I return to clinic I must consult health care provider and ask her that I want to know the results” (IDI respondent no 28; 46-years male)*

During FGDs, the HCPs expressed that they perform intensive follow up on adolescents and young adults who are not taking ARVs as prescribed; therefore, during this process they provide information on timing of HVL test some they understand but some do not.

One HCP said that:

*When we take the blood for viral load testing those adolescents with poor results we don’t sit just leave them, we have a system where those with good results talk with the poor to make them motivated and learn how they have made good adherence to have good results. (FGD respondent no 2;36 years’ nurse counsellor)*

### Category I: Information on HIV viral load results

We sought to know if AYA were informed about the meaning of the HVL results and observed that generally AYA and parents or guardians reported discussing HVL results with their HCPs. In addition, participants declared that the delay in results is no longer a significant problem like in the past few years. Moreover, participants feel that they need to have ownership over HIV treatment monitoring; asking for the HVL test results, the next HVL test appointment date, sometimes remind the HCP on the due date for HVL testing and discussing the meaning of HVL results**.** Principally, IDI participants expressed that HVL results are essential to them as an indicator of their treatment progress. One adolescent said:

“*In November last year, I was told my viral load results are low reading 14. I felt good and encouraged to continue taking my medicine. All I want is to be involved in my treatment,* (*IDI respondent 14; 19 years male)*

One youth said that:

*“For me most of the time when my clinic date approaches I must contact the provider and ask, please check my file if I have the check-ups, they say you don’t have, aa I want to know my health improvements, you don’t have the check-ups until next month”. (IDI respondent no 8;17-years female)*

### Category II: Interpretation of viral load results

Older adolescents (15–19 years), parents and guardians reported a good understanding of the meaning of viral load results, and they discussed the results with HCPs. On average, IDI participants reported that good results mean viruses are detected below 50 copies and above 50 copies indicate viruses are high in people with poor ART adherence.

*“I spent a lot of time with XX [providers name] he told me that the results show viral load is low, and asked If I face any challenges. He added that he would like to see this situation being sustained (IDI respondent no 3; 21 years male)*

Fifty-five percent of parents or guardians (5/9) reported discussing HVL results in the presence of their child, insisting that children or young adolescents with good results are congratulated and encouraged.

One parent said:

“*We receive results while together, she is told do you see how your result are good? She is encouraged to continue with school, try to eat well and avoid bad thoughts so that you can continue well”.* (*IDI respondent no 23; 47 years female)*

We noted that less often parents or guardians did not discuss their children or young adolescent HVL results. One reason mentioned was not having time to go to the clinic together.

*“I was not present when the results were given to him, he visits alone, he asks for transport I give him because I had to go to work. So, I give him transport and he visits alone”. (IDI respondent no 11; 43 years male)* Another parent said:

*The clinic called him but I did not get the time to talk to him and ask but I think they discussed with him about his CD4 results. I don’t know what they check, I did not ask him. (IDI respondent no 28; 46 years male)*

One youth declared that her high viral load resulted from treatment interruption, so when the HCP discussed the results, she knew the problem and corrected it. The subsequent HVL testing was good because she fixed the problem and improved her adherence.

*“At first, I was told the viruses are high, I was not taking my medicines because, when I travel I forget them at home. The nurse explained to me, if you don’t take your ARVs, things will be worse. After taking medicines very well, I repeated the test on the coming months and they told me you have low viruses” (IDI respondent no 24; 21- years female)*

We found a variation in understanding HVL results. One young adolescent did not know what his results were after testing. He assumes her aunt (guardian) knows the results. He said:

*“They don’t tell me; they tell my aunt. I come to clinic, I take medicine, the nurse writes for me the next appointment date and then I leave” (IDI respondent no 10; 11 years’ female)*

Similarly, one guardian reported experiencing no details of the HVL results for the adolescent he is caring for. Another guardian described not understanding the details as to why HVL is high while the child/adolescent has good adherence. The HCP did not discuss the details.

*“Sometimes it’s difficult to talk we just listen to what the provider has to say and then we leave. But that’s the sign that the progress is not good because we were collecting the medicine after two months and now we have been taken to where we came from (one month)”. (IDI respondent no 18; 45 years female guardian)*

***“*** *They mostly say you are continuing well, continue to use medicines correctly. Nothing else. (IDI respondent no 12; 37 years female parent)*

In contrast, during FGDs the HCPs expressed that within one week of receiving results from the testing laboratory. A recipient of care with high HVL ≥ 1000 copies/ml will be immediately called using a mobile and plan for a clinic visit to start EAC and the clients with results of below 1000 copies will not eb called but rather their results will be discussed when the client comes to their scheduled visit.

One HCP stated the following as a procedure for discussing VL results:

*“First of all after receiving results before disclosing them to clients we usually discuss as a team, that we are supposed to call the client, so we call him/her through telephone and after the preparation as a team of five to six people we sit with the client and welcome our client by taking away his fears and we tell him we called you because we have seen your results of viral load is above thousands so we want to talk to you in detail. We usually allow him to be open and we calm him down because most of the time they disclose the challenges they face. So, we deal with the challenges he/she mentions first by advising him to take meds on time and to stop unprotected sex. If he/she doesn’t travel with the CTC card we advise him to do so, so we usually advise him/he based on what he/she disclose to us.*

Importantly, HCPs insisted that they usually inform their clients of the importance of ART adherence on their health outcomes including good HVL results.

*“We providers make sure that our recipients of care should know that taking drugs and taking tests are two things which are interdependent. They should know why they are taking drugs. They should know after taking drugs we need to know the outcome. (FDG respondent, 43 years’ nurse counsellor)*

The HCPs described a prompt response to clients’ HVL results >1000copies/ml by calling them to come for initiating EAC, and those with HVL < 1000copies/ml will receive their results during their scheduled appointment.

*“So, within that week of receiving the results the recipient of care will be called the same day but, the problem we encounter is that some clients take a long time to come to the clinic. They can take two weeks or just come on his next scheduled visit. (FDG respondent 12;39 years nurse counsellor).*

HCPs explained that during a discussion with a client on high HVL, listening to the client’s challenges is vital to building a case for solving the challenges that lead to high HVL and the way forward. For example, one HCP explained that some adolescents and youths are disappointed when their results are not suitable while they have good adherence.

One quote from HCP:

*“Adolescents become disappointed because for them to take medicine at first was a hustle then when s/he takes it correctly and then you tell him/her that the viral load is still high. They become disappointed. Although education is provided but according to their age they can think that way. (FDG respondent, 46 years nurse counsellor)*

### Theme 3: Barriers or challenges to implementation of viral load testing

Overall, AYA participants reported that there had been no significant challenges or barriers in the HVL testing procedures in recent years. However, in the past 2–3 years, delays in receiving HVL results took about 2–3 months to obtain results and sometimes they had to repeat collecting the blood samples.

*“Testing is just normal I don’t see any problem for now. (IDI respondent no 8;17-years female)*

***“*** *I don’t know what happened because they took blood for the check-ups but after that they called me again to come for the check-ups. I told them I have already taken blood for the check-ups. They told me the laboratory did not provide the results, so I decided to come for the test again. (IDI respondent no 7;16-years male)*

*“Sometimes in school, exams date is the same with the clinic date. So, I am forced to tell the teacher and leave school for clinic and since the teacher knows my health status, she also knows my clinic date, so she keeps my exams and when I return I do it. (IDI respondent no 1; 12 years male)*

### Category I: Individual Involvement

During FGDs with HCPs, the implementation challenges reported were more client-related factors. The HCP follow the algorithm of HVL testing as indicated in the national guideline, but clients may not attend the scheduled EACs sessions for those with high HVL results. Most clients will explain reasons for not following the EAC schedule, including financial constraints to afford transport for frequent clinic visits. But HCP has noticed that clients who are given 3–6 months ARVs have challenges attending when they are recalled back for EAC initiation. HCP think that once a client has enough drugs/ ARVs, they do not come back even after counselling via mobile call conversation. As a result, some clients will refill ARVs at another facility while they are due for HVL testing as scheduled at the usual CTC. Another challenge is when a client travels for work or business, they request a three-month refill or come to refill one month before their scheduled visit date while their next HVL test also include HVL testing. Therefore, HCP reported that the guideline needs to indicate if the HVL test can be done one month before the due date.

HCP insisted that adolescents and youth in boarding schools, colleges and universities have no timely HVL testing. The reasons are being at school when it is their time for HVL testing and still in school. This challenge is improving for those attending Saturday’s youth clinics. However, HCP still reported about 30% of adolescents face conflicting timetables for their school activities, mainly exams. In addition, independent young adults also face untimely HVL testing as they explain that they cannot ask for permission from work or due to non-disclosure to their employers.

***“Another challenge I have observed is that children and adolescents, whether attending boarding school or day scholars, might have school exams scheduled on the same day as their viral load (HVL) testing appointments. Some even attend school on Saturdays, which makes it difficult for them to keep their HVL testing appointments. (FGD respondent no 9; 34 years ART nurse)”***

### Category II: Centralized HVL testing

HCP described that the centralized laboratory testing is operating smoothly. Still, when the machines break down, it becomes a disaster this service in mainly provided by one laboratory for all ART facilities in Dar es Salaam. In addition, HCP reported that they experienced three months’ delay in receiving HVL results when Temeke specialized laboratory faced a machine breakdown in the past two years. Consequently, clients with high HVL who require EAC and a second HVL will stay pending until services resume. Less frequently, HCP are required to repeat HVL sample collection due to known and unknown laboratory reasons. As a result, it becomes challenging to convince a client to repeat a blood sample collection.

*“In the past two to three years, it took even three or four months waiting for results. For an adolescent who has tried to change behavior s/he believes that the viral load test result will be good but you keep him/her waiting for three or four months. This group is usually emotional so, the issue of waiting for so long may take her/him to the former state of high viral load. (FDG respondent no 1; 49 years nurse counsellor)*

*“Very few times we are told that the test reagent is out of stock or the system is down, you will get the results back on another day. Those are the usual things that happen although not so frequent. (IDI respondent no 17; 42 years female parent).*

*“The majority of clients missed the clinic because they were scared of being infected with the coronavirus. Others were out of Dar so traveling back was challenging. The majority of the clients were eligible for viral load testing but it was difficult to get them”. (FDG respondent no 4; 37-years clinician)*

## Discussion

**Our qualitative study involving adolescents and young adults living with HIV, parents or guardians of young adolescents living with HIV, and healthcare providers (HCPs) in urban Tanzania found that the majority of adolescents recognized and accepted HIV treatment as a lifelong commitment.** Notably, AYA in our study were mainly urban residents, with 70% enrolled in school or college. About 10% of adolescents interviewed reported to be tired of taking ART and wished HIV to become a curable disease. We noted treatment fatigue being one of the reasons adolescents living with HIV (ALHIV), especially for those who have been on ART since childhood, may have poorer outcomes than HIV-infected adults [27], or emotional tiredness from taking ARVs [28]. Comparable to South African adolescents, where 24.6% reported elevated levels of fatigue which was associated with increased viral loads [29]. Other studies have reported that the consequences of treatment fatigue include treatment interruption, decreased adherence, non-adherence, and discontinuation of ART [28]. Therefore, it is crucial to explore potential interventions to reduce treatment fatigue and increase adherence, such as introducing novel antiretroviral agents known as long-acting injectable ART in our setting. Meanwhile, the implementation of quality psychosocial support is paramount in the package of adolescent-friendly services to cover such situations [30,31]. Notably, we found that 32% of AYA wish to receive injectable ARVs to improve adherence and reduce stigma. Similarly, in **the** USA AYA showed interest with Long acting ART [32], while in Tanzania and the Dominican Republic adult female sex workers living with HIV revealed significant positive attitudes towards long acting injectable ART [33]. In contrast, open label extension trials suggested that long acting injectable ART was highly acceptable, about 97% of participants decided to remain on injectable ART [34]. Acceptability and positive attitude about long acting injectable ART vary in different populations of PLHIV however, similar potential benefits are reported. Importantly, clinical trials of long acting injectable ART have been conducted and shown to be effective in improving ART outcomes [35]. Therefore, long-acting ART is a potential intervention that is alternative to oral ART among adolescents that could potentially benefit from improving adherence. Given that adolescents present unique challenges that increase the risk of non-suppressed viral loads, long-acting injectable ARVs warrants monitoring in AYA who are at risk of ART failure as one of the targeted interventions to improve HIV-related treatment outcomes.

**Our findings reveal that most IDI participants reported understanding the benefits of testing for viral load because it helps to know if HIV treatment is working and reflects if a person is taking medicine correctly.** In addition, parents or guardians, older adolescents, and young adults described ownership in the follow-up of HVL testing by asking healthcare providers about the details of HVL results. In contrast, almost all AYA enrolled in a qualitative study in Zimbabwe did not understand why viral load monitoring is done [36]. In the current study, about 60% of participants reported discussing HVL results with their healthcare providers. Our findings are different for AYA with undetectable viral load in Zimbabwe, where the results were not discussed with AYA but documented in their clinic file [36]. These findings underscore the importance of the recipient of care following up the gold standard for accurate monitoring of ART and detection of virologic failure [6]. Knowing the viral load status is crucial to keep HIV suppressed and individuals who sustain viral load suppression have a very minimal risk of sexually transmitting the virus to a partner who is HIV-negative [37,38]. Nevertheless, understanding viral load status encourages PLHIV to have self-strategies to improve adherence. Importantly, unsuppressed people should know their status and should be provided with strategies or interventions to achieve viral suppression. Therefore, it is paramount for HCP to ensure that adolescents understand their viral load test results and their interpretation, which will guide further management plans. Furthermore, we observed that suppressed viral load results make AYA feel excited about their future as opposed to a similar age group in Africa who did not understand how viral suppression could positively impact their social and relational lives [36]. Thus, AYA become confident knowing the viruses are controlled, which encourages and motivate them to continue with good adherence.

On the other hand, 32% of AYA reported experience of being disappointed with high viral load results as they reported having good ART adherence. High viral load in the context of good ART adherence may be attributed to the development of drug resistance [39]. Such clients warrant HIV drug resistance testing to enable clinicians to select effective drugs and improve viral suppression.

Non-disclosure of HIV-positive status has been shown to be associated with non-adherence to ART among AYA [40,41]. Less than 20% of AYA did not disclose their HIV-positive status to immediate family members or relatives affected adherence to ART, similar to other studies [41,42]. AYA who are in college or university and stay in hostels reported to face a challenge to adherence due to non-disclosure to their colleagues and sharing of bedrooms (lack of privacy) with college/ university roommates. In addition, disclosure of HIV status to others beyond a family is challenging because there is a sense of guilt in sexually infected adolescents, a tendency to blame parents if vertically affected, and fear of stigma [42,43]. Also, disclosure of HIV-positive status to a new spouse in young adults, particularly girls and young women, is challenging [44]. One study in South Africa examined disclosure to sexual partners and reported that married women were reported to be three times more likely to disclose their status to their partners relative to single women [44]. Importantly, since young women desire to have spouses and children [45]; the need to support them for HIV positive status disclosure to their sexual partners, relatives, and their social networks using HIV disclosure toolkits and receiving sexual reproductive health services is imperative. Studies have reported that PLHIV who disclosed their HIV status are freely able to take their drugs had better family and social support leading to a reduction in depression, improved health outcomes, and good ART adherence [40,46]. Therefore, psychosocial support, peer support and family support are key components to assist adolescents and young adults who have disclosure issues.

More than half (58.6%) of the study participants during IDI knew their HVL testing schedules from individual client consultation or through collective health education; this was the primary facilitator of the implementation of HVL testing. In addition, IDI participants reported no significant HVL testing challenges they were facing compared to the past few years. However, HCP expressed challenges in HVL testing for AYA in boarding schools and colleges or universities because their school activities may have a similar schedule with clinic visits or programs for HVL testing. Thus, delays in HVL testing as required indicate delays in detecting VF that may also result in drug resistance. As a consequence, a significant drawback for timely EAC. Similarly, in Kenya. boarding school or higher-level education environment was reported to facilitate HIV care and treatment challenges in AYA [47]. These challenges include HIV status non-disclosure to peers, lack of support with adherence, fear of stigma, and limited privacy [47]. Therefore, HIV national programs must look at school interventions necessary to transform the school environment to become supportive of eliminating the challenges or barriers that hinder good ART outcomes.

Another HVL monitoring implementation challenge is missing clinic appointments in AYA receiving three to six multi-month ARV dispensing (MMD), they do not return when recalled for EAC initiation. Consequently, delays in the initiation of EAC and untimely second HVL test occur in the EAC cascade. Our findings are similar to a quantitative study in Nigeria where the uptake of HVL testing at 6 and 12 months after ART initiation was low, about 70% of the eligible adolescents and adults had missing HVL testing [48]. However, among those tested HVL no significant differences in viral suppression or undetectable HVL results at 6 months and at 12 months between newly enrolled patients on MMD and those not on MMD [48]. Notably, in Tanzania, the scale-up of 3–6 months’ multi-month dispensing was adopted as recommended by the WHO in Tanzania pending evidence of feasibility and effectiveness to the healthcare system and recipients of care. Therefore, the national ART program must recognize the need to evaluate implementation facilitators and barriers concerning the benefits of a six MMD on viral suppression in populations at risk for ART failure.

The last challenge reported by HCPs that few independent young adults travel for work or business; they request three months of ARV supply or come to refill one month before their clinic appointment while HCP are unsure to take HVL sample at this time or not. Notably, the HCPs needs guidance on whether they can take samples for HVL testing one month before the recommended time because the national guideline is silent on this situation. HCPs aim to reduce missing appointments for HVL testing for those with a history of good ART adherence and previous viral suppression results known as stable on ART clients.

### Implications to improve HIV programming and treatment

Our study findings have implications for improving HIV care for adolescents and youth by promoting a patient-centered approach empowering them in HIV treatment and care ownership to support effective treatment. This would help promote positive ART outcomes. Importantly, strengthening the existing adolescent and youth-friendly clinics by enhancing their engagement in treatment and care, therefore promoting viral suppression. Furthermore, optimizing HIV viral load testing coupled with a clear understanding of results interpretation will enhance ART adherence and motivation in continuum of care ultimately fostering viral suppression and, establishing tailored interventions for those with unsuppressed viral loads.

### Strength and limitations

The investigators gathered rich data through in-depth interviews with AYA to understand more about the journey of the recipient of HIV monitoring care, and this information served as the basis for working out the narratives within interviews. In addition, the AYA insights set the ground for a potential to make well-informed decisions in developing strategies to improve HVL monitoring in AYA in the direction of achieving the third 95 UNAIDS target (95% of all people receiving antiretroviral therapy to have VS).

We conducted our study in an urban setting, and caution should be taken for generalizing these results to other HIV cohorts, especially in rural settings. Notably, study investigators could not interview young adolescents (10–14 years) who had not fully disclosed their HIV status because it would interfere with the ongoing disclosure process. The consequences of premature disclosure by our study interviews could increase the risk of mental and behavioral issues, family conflicts and perceptions of stigma. We mitigated this by interviewing the parents or guardians of these young adolescents as they come along with them to the clinic and, therefore, have experience in the HIV cascade of care. Also, we conducted FDGs among HCPs to provide additional information on experiences and explore complex situations in HVL monitoring.

### Conclusions

More than half of AYA expressed a desire for ownership in their HIV treatment monitoring; they understood and discussed the meaning of their viral load results with HCPs. Additionally, AYA reported facing non-frequent challenges in HVL testing however, HCPs described challenges for implementing viral load testing include missing appointments for AYA and clients on three to six MMD of ARVs. Therefore, we recommend that the national ART program needs to strengthen the involvement and communication to AYA as the central part of HVL monitoring by motivating them to play a vital role towards participation in overall lifelong treatment with good outcomes.
